# Effect of CpG-Oligonucleotide in Enhancing Recombinant Herpes Virus of Turkey-Laryngotracheitis Vaccine-Induced Immune Responses in One-Day-Old Broiler Chickens

**DOI:** 10.3390/vaccines11020294

**Published:** 2023-01-29

**Authors:** Carissa Gaghan, Matthew Browning, Aneg L. Cortes, Isabel M. Gimeno, Raveendra R. Kulkarni

**Affiliations:** Department of Population Health and Pathobiology, College of Veterinary Medicine, North Carolina State University, 1060 William Moore Drive, Raleigh, NC 27606, USA

**Keywords:** infectious laryngotracheitis, CpG-ODN, adjuvant, immune response, chickens

## Abstract

Infectious laryngotracheitis (ILT) is an economically important disease of chickens. While the recombinant vaccines can reduce clinical disease severity, the associated drawbacks are poor immunogenicity and delayed onset of immunity. Here, we used CpG-oligonucleotides (ODN) as an *in ovo* adjuvant in boosting recombinant herpesvirus of turkey-laryngotracheitis (rHVT-LT) vaccine-induced responses in one-day-old broiler chickens. Two CpG-ODN doses (5 and 10 μg/egg) with no adverse effect on the vaccine-virus replication or chick hatchability were selected for immune-response evaluation. Results showed that while CpG-ODN adjuvantation induced an increased transcription of splenic IFNγ and IL-1β, and lung IFNγ genes, the IL-1β gene expression in the lung was significantly downregulated compared to the control. Additionally, the transcription of toll-like receptor (TLR)21 in the spleen and lung and inducible nitric oxide synthase (iNOS) in the spleen of all vaccinated groups was significantly reduced. Furthermore, splenic cellular immunophenotyping showed that the CpG-ODN-10μg adjuvanted vaccination induced a significantly higher number of macrophages, TCRγδ+, and CD4+ T cells as well as a higher frequency of activated T cells (CD4+CD44+) when compared to the control. Collectively, the findings suggested that CpG-ODN can boost rHVT-LT-induced immune responses in day-old chicks, which may help in anti-ILT defense during their later stages of life.

## 1. Introduction

Infectious laryngotracheitis (ILT) is a highly contagious respiratory infection in chickens caused by an alphaherpesvirus, known as ILT virus (ILTV). Outbreaks of ILT, particularly in the broiler flocks, cause significant economic losses due to increased mortality and carcass condemnation coupled with decreased bird performance [1]. Part of the problem in these flocks is also that the periodic live vaccine virus spills over from vaccinated breeder facilities with a rapid development of clinical disease in virus-exposed broiler flocks [2]. Current commercial vaccines are of two types: live attenuated or modified vaccines, namely the chicken-embryo origin (CEO) or tissue-culture-origin (TCO) vaccine, and recombinant vaccines, namely the herpesvirus of turkeys (rHVT-LT) or fowl poxvirus (rFPV-LT) vaccines [3,4]. Of these vaccines, the live attenuated vaccines, specifically the CEO vaccine, can effectively prevent clinical disease and mortalities. However, the major disadvantage associated with this vaccine is that the vaccination can cause increased virulence of the virus through bird-to-bird passage, resulting in increased disease severity and deaths [2,5]. Recombinant ILT vaccines (rILT), on the other hand, approved for *in ovo* delivery as safe and devoid of virulence enhancement issues, are shown to reduce the severity of clinical disease and help improve bird performance [4]. For example, we have previously shown that *in ovo* immunization with rHVT-LT or rFPV-LT vaccines in chickens can significantly reduce tracheal-virus replication as well as clinical signs along with an improvement in body weight gain [6,7]. However, when compared with the CEO vaccination, the immunogenicity and level of protection offered by these vaccines was poor, the onset of immunity was delayed, and shedding of challenge virus was not completely curtailed [4,6,8,9,10]. These studies indicated that adjuvantation of rILT vaccines may help overcome some or all these drawbacks associated with the recombinant vaccines.

In poultry, the use of toll-like receptor (TLR) adjuvants has been successfully tested in boosting vaccine-induced immunity. TLRs are a family of pattern-recognition receptors expressed by immune cells, which upon binding to microbial ligands, initiate key host immune defense against infectious agents [11]. TLR9 in mammals and TLR21 in birds recognize microbial DNA containing unmethylated cytosine–guanosine deoxynucleotide (CpG) oligodeoxynucleotides (ODN) [12]. Medical research shows that CpG-ODN possess immunostimulatory or immunomodulatory properties and hence, these ODN molecules are used as therapeutic agents or as vaccine adjuvants [13,14]. In chickens, we and others have previously shown that CpG-ODN can be used as an immunostimulatory molecule or as a vaccine adjuvant to boost immunity against bacterial and viral diseases [15,16,17,18,19]. For example, CpG-ODN treatment of chick embryo fibroblasts can reduce Marek’s disease virus (MDV) replication [16], while *in ovo* administration of CpG-ODN has been shown to inhibit replication of ILTV and infectious bronchitis virus (IBV) in chickens post-hatch [20,21]. However, data related to the use of CpG-ODN as an *in ovo* adjuvant for rILT vaccine are currently lacking, which was the objective of the present investigation.

Here, we have used CpG-ODN to adjuvant rHVT-LT vaccine for *in ovo* administration and assessed vaccine-induced immune responses in one-day-old chicks post-hatch. To this end, we first performed dose-range-finding experiments to select CpG-ODN doses that had no adverse effect on the vaccine-virus replication and chick hatchability. Next, we evaluated the vaccine-induced immune responses by measuring immune-gene expression in the spleen and lung tissues as well as splenic cellular immunophenotypes.

## 2. Materials and Methods

### 2.1. Animals

Fertile eggs, Ross 708-line broiler, (source: Aviagen Inc. Huntsville, AL, USA) were used in the dose-optimization, immune-gene-expression and cellular-immunophenotyping experiments. About 15–20 embryonated eggs were randomly assigned to four treatment groups that consisted of (1) vehicle-only (sham) control, (2) rHVT-LT only, (3) rHVT-LT + CpG-ODN 5 μg, and (4) rHVT-LT + CpG-ODN 10 μg doses. Embryonated eggs were administered via the amniotic route with different treatments on the embryonic day (ED) 18 and hatchability was assessed around day 21. For collecting tissues, the day-old chicks were euthanized using carbon dioxide gas and all the animal experiment protocols used in this study were approved by the North Carolina State University Institutional Animal Care and Use Committee (IACUC, protocol # 21-203).

### 2.2. Vaccines

Commercial rHVT-LT vaccine expressing the ILTV glycoprotein B (gB) and UL-32 proteins was obtained from Ceva Animal Health Inc. (Lenexa, KS, USA). The synthetic CpG-ODN (ODN 2007, InvivoGen, San Diego, CA, USA) used in the present study was a class B CpG ODN containing a full phosphorothioate backbone with one or more CpG dinucleotides. The lyophilized powder of CpG-ODN was dissolved in endotoxin-free water and stock solutions of 10 mg/mL were prepared. The CpG-ODN was mixed with rHVT-LT vaccine in a volume of 100 µL/egg for *in ovo* administrations.

### 2.3. Virus Titration and Hatchability

**Figure 1:**
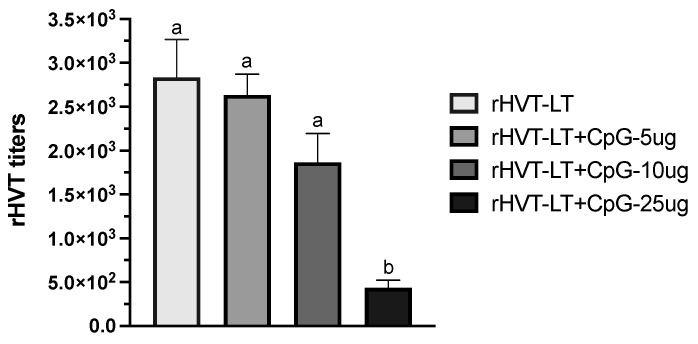
Effect of CpG-ODN on rHVT-LT vaccine-virus replication in vitro. Monolayer cultures of chicken embryo fibroblasts (CEFs) were infected with one dose of vaccine and with three serial 10-fold dilutions. Vaccine ± CpG-ODN doses (5, 10, and 25 µg) were titrated in triplicate and the growth of the virus was determined through assessing the cytopathic effect on CEFs using an inverted microscope. Titration was evaluated through counting the number of plaque-forming units (PFUs). Different letters above the standard-error-of-mean bars indicate significant difference (*p* < 0.05) between the groups.

Vaccine-virus titration was conducted by plaque assay as reported previously (Lopez Juan de Abad et al.; 2019). Secondary chicken embryo fibroblasts (CEFs) were seeded with Leibovitz/McCoy medium modified with glutamine media and 4% calf serum for 24 h until a confluent monolayer formed. The monolayer was supplemented with 2% calf serum, changed every other day for the duration of the titration, and cells were incubated at 37 °C with 5% CO_2_. Briefly monolayer cultures of CEFs were infected with one dose of vaccine (which was reconstituted as per the manufacturers recommendations) and with three serial 10-fold dilutions. Vaccine ± CpG-ODN doses (5, 10, and 25 µg) were titrated in triplicate and the growth of the virus was determined through assessing the cytopathic effect on CEFs using an inverted microscope. Titration was evaluated through counting the number of plaque-forming nits (PFUs). Post-ED18 *in ovo* inoculations, the chicks that hatched in each of the treatment groups were counted and the hatchability was calculated and depicted in Figure 1.

### 2.4. Immune-Gene Expression

Spleen and lung were collected from each of the treatment groups (n = 8) in RNAlater solution (Invitrogen, Carlsbad, CA, USA) from chicks on day of hatch and stored at −80 °C until processing. Total RNA was extracted using a Bead Ruptor Elite Bead Mill Homogenizer (OMNI International, Kennesaw, GA, USA) using 1.4 mm ceramic beads (OMNI International, Kennesaw, GA, USA) suspended in TRIzol reagent (Invitrogen, Carlsbad, CA, USA) according to the manufacturer’s protocol before being treated with a DNA-free Kit (Invitrogen, Carlsbad, CA, USA). Following this, cDNA synthesis was performed with 1000 ng of purified RNA using a High-Capacity RNA-to-cDNA Kit (Applied Biosystems, Waltham, MA, USA), according to the manufacturer’s recommended protocol. The resulting cDNA was subsequently diluted 1:10 in nuclease-free water for real-time PCR analysis. Quantitative real-time reverse-transcriptase PCR using SYBR Green was performed on diluted cDNA using a QuantStudio 6 Flex System and QuantStudio Real-Time PCR Software (Applied Biosystems, Waltham, MA, USA). Briefly, each reaction involved a pre-incubation period of 50 °C for two minutes followed by 95 °C for two minutes, followed by 35–45 cycles of 95 °C for 10 s, 55–64 °C for 5 s, depending on the primer’s binding suitability, and the elongation step was 72 °C for 10 s. Subsequent melt-curve analysis was performed by heating to 95 °C for 15 s, cooling to 60 °C for 1 min, and heating to 95 °C for 15 s. Primers for the amplification of interferon (IFN)γ, interleukin (IL)-12, IFNβ, IL-1β, inducible nitric oxide synthase (iNOS), and TLR21 genes were synthesized by Integrated DNA Technologies (Coralville, IA, USA), and the primer sequences are given in Table 1. Expression levels of target genes were calculated relative to the stably expressed reference gene, β-actin [22], using the relative gene expression method [23,24].

### 2.5. Flow Cytometry

Single-cell splenocyte preparations (n = 8 per group) were made following a protocol described previously [25]. Briefly, the single cells were plated on 96-well round-bottom plates with each well containing 10^6^ cells in 100 μL FACS buffer (PBS with 1% BSA). Primary antibodies were added to each well (0.5–1 μg/10^6^ cells). Cells were stained in two different panels of antibody staining due to the paucity of having chicken antibody reagents available in multi-color formats. All anti-chicken antibodies were purchased from Southern Biotech Inc., Birmingham, AL, USA, and were of mouse origin, and their respective clones are given the parenthesis below. The first panel used antibodies against mannose receptor C1-like B (MRC1L-B) (monocyte/macrophage lineage, clone KUL01), γδTCR (TCR-1), and the second panel used anti-CD3 (CT-3), CD4 (CT-4), CD8 (CT-8 recognizing CD8α chain), and CD44 (AV6). In both panels, cell viability dye, Live/Dead Near IR (Invitrogen, Calsbad, CA, USA) was used to exclude dead cells. Stained cells along with the single stain compensation and staining controls were washed and fixed with 4% paraformaldehyde before data acquisition using LSR-II flow cytometer (BD Biosciences, Franklin Lakes, NJ, USA). Data analysis was carried out using the FlowJo software (Tree Starr, Ashland, OR, USA). The gating strategy included exclusion of doublet cells through forward and side scatter plotting using the area (A), height (H), and width (W) followed by gating on live cells Briefly, analysis of all the samples was performed on the basic ‘live cell’ gate and the cells expressing mannose receptor C1-like B (MRC1L-B) (monocyte/macrophage lineage, clone KUL01) were gated as macrophages and the TCRγδ+ cells as γδT cells. To determine the frequencies of T cell subsets, CD4+, CD8+, and double-positive (DP) cells, the live CD3+ population was used as the backbone gate. To evaluate T cell activation, CD44 was used as the target-cell activation marker such that the live CD44+ cell population served as the primary population from which the CD3+ cells (T cells) were gated and further analyzed for the populations expressing CD4, CD8, or both (DP cell) markers. 

### 2.6. Statistical Analysis

All the data pertaining to virus titration, immune-gene expression, and immunophenotyping were analyzed using GraphPad Prism V9.4 (GraphPad software, San Diego, CA, USA). Depending on the data distribution, parametric test, one/two-way ANOVA followed by Turkey’s multiple comparisons test, or the nonparametric test (Kruskal–Wallis), were used [26]. Data were presented as mean ± standard error of the mean (SEM) and the results were considered statistically significant at *p* < 0.05.

## 3. Results

### 3.1. Virus Titration and Hatchability

In order to determine the effect of different doses of CpG-ODN on the vaccine virus (rHVT) replication in vitro, virus titration was performed. As shown in Figure 1, of the three doses (5, 10, and 25 µg) adjuvanted with the vaccine, the CpG-ODN at 25 µg treatment of CEF led to reduced (*p* < 0.05) virus titers when compared to other CpG-ODN concentrations as well as the rHVT-only group. Considering the toxicity associated with the 25 µg dose, further hatchability testing included only the 5 and 10 µg CpG-ODN adjuvated doses.

Chick hatchability in each of the treatment groups was calculated as the number of hatched chicks as a percent of total number of embryonated eggs that were *in ovo* inoculated. As depicted in Table 2, each of the sham and rHVT-only groups had 13 of the 15 (86.7%) embryonated eggs hatch post-inoculation, while those receiving the rHVT adjuvanted with 5 and 10 µg had a hatchability of 93.3% and 80%, respectively. Although the group receiving CpG-ODN (10 µg) had a relatively reduced hatchability when compared to other treatments, no statistically significant changes were observed between the groups. These results indicated that the CpG-ODN adjuvanted at the concentrations of 5 and 10 µg were safe for *in ovo* administration to allow further evaluation of vaccine ± adjuvant-induced immune responses in one-day-old chicks.

### 3.2. Immune-Gene Expression

**Figure 2:**
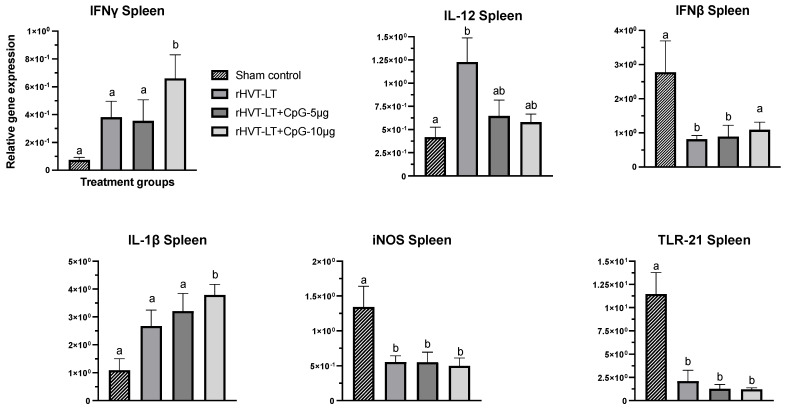
Effect of CpG-ODN adjuvantation on the immune-gene expression in the spleen. Recombinant HVT-LT (rHVT-LT) vaccine was adjuvanted with either 5 μg or 10 μg of CpG-ODN and administered *in ovo* on the day 18 of embryonation (ED18) via the amniotic route. Spleens from the day-old broiler chicks were collected in RNAlater for RNA extraction and cDNA synthesis. Real-time PCR to quantify the expression of IFNγ, IL-12, IFNβ, IL-1β, iNOS (inducible nitric oxide synthase), and TLR21 genes was performed along with the housekeeping gene (β-actin). The expression levels are shown as relative to β-actin. Different letters above the standard-error-of-mean bars indicate significant difference (*p* < 0.05) between the groups.

**Figure 3:**
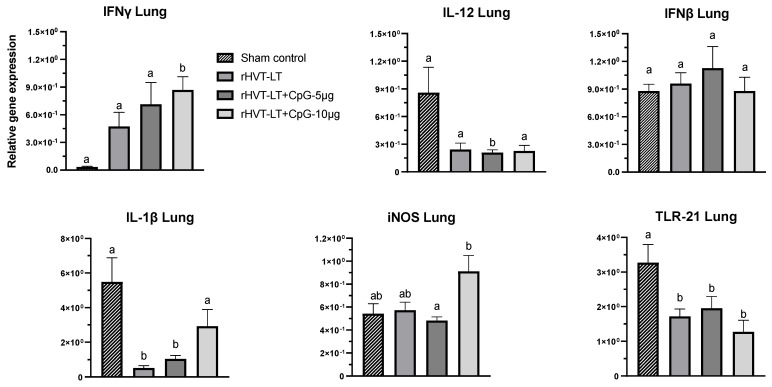
Effect of CpG-ODN adjuvantation on the immune-gene expression in the lung. Recombinant HVT-LT (rHVT-LT) vaccine was adjuvanted with either 5 μg or 10 μg of CpG-ODN and administered *in ovo* on the day 18 of embryonation (ED18) via the amniotic route. Lung tissues from the day-old broiler chicks were collected in RNAlater for RNA extraction and cDNA synthesis. Real-time PCR to quantify the expression of IFNγ, IL-12, IFNβ, IL-1β, iNOS (inducible nitric oxide synthase), and TLR21 genes was performed along with the housekeeping gene (β-actin). The expression levels are shown as relative to β-actin. Different letters above the standard-error-of-mean bars indicate significant difference (*p* < 0.05) between the groups.

To evaluate the effect of CpG-ODN adjuvanted rHVT-LT vaccines on the local as well as the systemic immune response, the spleen and lung samples were collected from day-old chicks and the expression of TLR21, IL-12, IL-1ß, IFNγ, IFNβ, and iNOS was quantified and the results are depicted in Figure 2 (spleen), and Figure 3 (lung).

As depicted in Figure 2, the splenic expression of IFNγ and IL-1ß in chicks receiving rHVT-LT + CpG ODN-10 ug was increased (*p* < 0.05) compared to other treatments. There was also a statistically non-significant increase in the expression of these cytokines observed in chicks administered with rHVT-LT or rHVT-LT + CpG ODN-5 µg when compared to the sham control group. The IL-12 transcription in the rHVT-LT group was also found increased (*p* < 0.05) compared to the sham treatment, while no significant changes were observed between the vaccinated groups. The expression of TLR21 and iNOS genes in all the treatment groups was decreased (*p* < 0.05) when compared to the sham group. Additionally, the IFNβ transcription in the birds receiving rHVT-LT or rHVT-LT + CpG ODN-5 ug was found reduced (*p* < 0.05) compared to control; however, no significant changes were observed between the rHVT-LT + CpG ODN-10 ug and sham groups. 

In the lung (Figure 3), the expression of IFNγ in the chicks receiving rHVT-LT + CpG ODN-10 ug was higher (*p* < 0.05) than the rest of the treatment groups, while the iNOS transcription in these birds was also found increased (*p* < 0.05) when compared to those administered rHVT-LT + CpG ODN-5 ug. Like in the spleen, the TLR21 expression in the lung of all the treatment groups was found reduced (*p* < 0.05) in comparison to the sham, while the transcription of IL-1β in the rHVT-LT or rHVT-LT + CpG ODN-5 ug groups was decreased (*p* < 0.05) than the groups that received sham or rHVT-LT + CpG ODN-10 ug. Furthermore, the birds receiving rHVT-LT + CpG ODN-5 ug also had reduced (*p* < 0.05) expression of IL-12 than the rest of the groups. However, no significant changes in the expression of IFNβ was observed between the treatment groups.

### 3.3. Immunophenotyping

**Figure 4:**
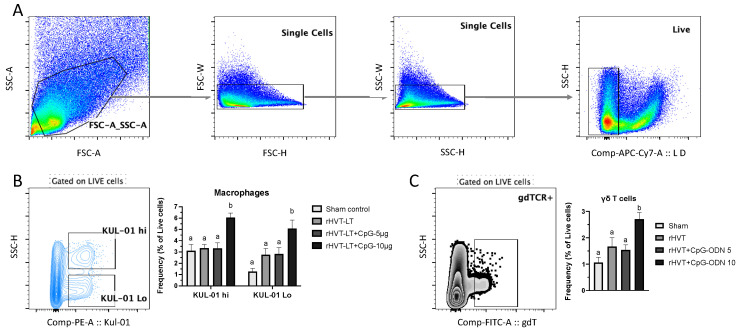
Effect of CpG-ODN on macrophage responses in one-day-old chicks. Single-cell splenocyte suspensions were prepared to obtain mononuclear cells and stained with anti-chicken monoclonal antibodies against KUL-01 (macrophage/monocyte lineage marker) and TCRγδ receptor. (**A**) Basic gating strategy showing representative analysis plots to exclude doublets from the populations and gating on live cells. (**B**) Live cells were gated to obtain macrophage populations and representative plot showing KUL-01 hi and KUL-01 Lo cells along with the bar chart depicting frequencies found across the treatment groups. (**C**) Live cells were gated to obtain TCRγδ+ populations and representative plot showing γδT cells along with the bar chart depicting frequencies found across the treatment groups. Different letters above the standard-error-of-mean bars within each of the data set graphs indicate significant statistical difference (*p* < 0.05) between the groups.

To evaluate the vaccine ± adjuvant-induced cellular response in the spleen, the frequency of macrophages and γδT cells as well as conventional T cell subsets (CD3+CD4+, CD3+CD8+, CD3+CD4+CD8+ referred to hereafter as CD4+ T cells, CD8+ T cells, and DP cells, respectively) was measured using flow cytometry. Additionally, using CD44 as an activation marker for T cells [27], we determined the cellular activation status of CD4+ T, CD8+ T, and DP cells. Figure 4A depicts the general gating strategy used to include singlets and live cells for an accurate analysis of these cell types. 

The analysis of macrophage populations based on their expression of the lineage marker, KUL-01 revealed two distinct populations as KUL-01 hi and KUL-01 Lo as shown in Figure 4B. The cellular frequencies of both populations were found increased (*p* < 0.05) in the group receiving rHVT-LT + CpG ODN-10 ug when compared to the rest of the groups. Additionally, those birds receiving rHVT-LT + CpG ODN-10 ug also had higher (*p* < 0.05) frequencies of γδT cells than the rest of the groups (Figure 4C). 

**Figure 5:**
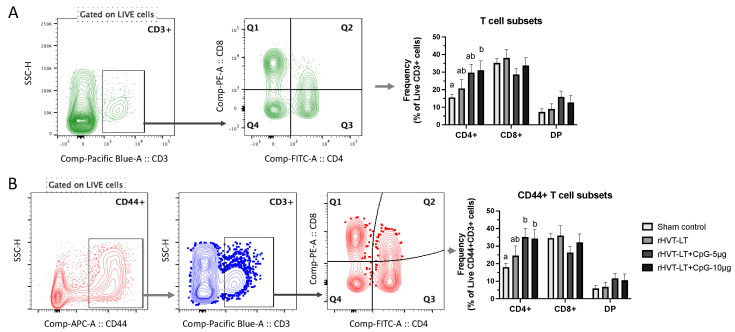
Effect of CpG-ODN on T-cell responses in one-day-old chicks. Single-cell splenocyte suspensions were prepared to obtain mononuclear cells and stained with anti-chicken monoclonal antibodies against CD3, CD4, CD8, and CD44 markers. The basic gating strategy included excluding doublets from the populations and gating on live cells. (**A**) Representative plot showing CD3+ gated population and a quadrant plot gated on CD3+ cells showing T cell subsets, CD4+ (Q3), CD8+ (Q1), and double-positive (DP) (Q2) cells along with the bar chart depicting their cellular frequencies. (**B**) Representative plots showing cells expressing CD44 gated on live cells to obtain CD3+ cells followed by quadrant gating showing T-cell subsets, CD44+CD4+ (Q3), CD44+CD8+ (Q1), and CD44+ DP (Q2) cells along with the bar chart depicting their cellular frequencies. Different letters above the standard-error-of-mean bars within each of the data set graphs indicate significant statistical difference (*p* < 0.05) between the groups.

The T cell analysis, as shown in Figure 5A, showed that the vaccination with rHVT-LT + CpG ODN-10 ug led to increased (*p* < 0.05) CD4+ T cell frequency when compared to the sham group, while no significant differences were observed between the vaccinated groups. However, a numerical increase was evident in the CD4+ T cell frequency in chickens receiving rHVT-LT + CpG ODN-10 ug compared to rHVT-LT group. Furthermore, the groups receiving adjuvanted vaccines (rHVT-LT + CpG ODN-5 or 10 µg) had higher activated (CD44+) CD4+ T cell frequencies when compared to the sham vaccinated group. Although a numerical increase in the CD44+CD4+ T cells was evident in the adjuvated vaccine groups when compared to rHVT-LT group, this difference was statistically not significant. No significant cellular changes in CD8+ T, DP, CD8+CD44+, or DP+CD44+ T cell frequencies were observed between vaccinated and sham control groups.

## 4. Discussion

Considering the increasing incidence of ILT in broiler flocks in recent years, which is negatively impacting the poultry-industry economy, there is an urgent need for safe, efficacious, and *in ovo* administrable vaccines. While the rILT vaccines are safe for *in ovo* use and effective in reducing clinical disease, we and others have previously found that the protection is not as effective as the one induced by CEO water vaccination [6,9,10,28]. The present study hypothesized that adjuvanting rHVT-LT vaccination *in ovo* with a well-characterized TLR adjuvant such as the immunostimulatory CpG-ODN molecule (TLR21 agonist) can boost immune responses in chickens as early as one day of age. Through experiments that included CpG-ODN dose optimization followed by immune-response evaluation in day-old chicks post-hatch, our findings yielded three pieces of information: CpG-ODN adjuvantation of rHVT-LT vaccine can (1) boost T helper-1 (Th1) immune response, as determined by the immune-gene expression and cellular immunophenotyping; (2) enhance splenic macrophage and γδT-cell responses, as a measure of innate immune function; and (3) induce an inflammatory response that seems spatially (lung and spleen) regulated. 

An effective Th1-type of immune response during viral infections plays a pivotal role in the clearance of viruses, including ILTV [20,29]. The Th1 component mainly comprises of CD4+ T cells that help CD8+ T, IgM+ B, and several other cell-types through the secretion of cytokines, of which IFNγ plays a critical role in the antiviral host defense. The present study observed that CpG-ODN (10 μg) adjuvantation could not only significantly augment CD4+ T cell frequency in the spleen but also induce enhanced activation of these cells, as determined by their expression of the CD44 molecule. It is noteworthy here that CD44 is an activation adhesion receptor involved in the T cell migration between the lymphoid tissues and site of infection [27]. Previous investigations studying protection against avian viruses, including ILTV have demonstrated the role of CD4+ T cells in antiviral immunity [30,31,32]. Importantly, studies investigating the role of CpG-ODN in host defense against ILTV and other pathogens have also indicated an immunostimulatory effect of this TLR21 agonist in augmenting CD4+ T-cell response [20,33]. Furthermore, rHVT-LT adjuvanted with CpG-ODN (10 µg) in the present study also led to a significant transcription of IFNγ gene in both spleen and lung tissues. Previous studies also have reported the important role of IFNγ in anti-ILTV defense in chickens [31,34,35] as well as *in ovo* CpG-DNA-mediated increased induction of this cytokine in ILTV or infectious bronchitis virus (IBV)-challenged 3-day-old chickens [20,33]. Considering the present study observations, it seems likely that the source of IFNγ in the CpG-ODN + rHVT-LT-administered chickens could have been CD4+ T cells [36] and that the CpG-ODN possessed Th1-adjuvant effects, which are critical in anti-ILT immunity. However, the present study did not find any significant changes in the CD8+ T cell frequency or their activation status between the treatment groups. This observation suggests that either the CpG-ODN effects on this cell-type may appear at a later timepoint of the chick’s life [29] or the observed effects are tissue-specific [20]. Collectively, our analyses of T cells and IFNγ gene expression suggests that CpG-ODN, when used as an adjuvant *in ovo*, can induce Th1-mediated CD4+ T-cell response, which may favor anti-ILT immunity in chickens. It is noteworthy here that *in ovo* adjuvantation of CpG-ODN with HVT has been recently shown to improve the vaccine efficacy in reducing the MDV-induced tumor incidence in chickens [37]. To this end, further studies are currently underway in our laboratory to evaluate and report if *in ovo* CpG-ODN (10 µg)-adjuvantation can enhance the protective ability of rILT vaccines in broiler chickens against an ILTV challenge at 14 and 28 days of age, as well as augment tissue-specific immune-gene expression and cellular responses.

Avian host innate defense plays a critical role in the initial antiviral combat and the two important cell types in this context are macrophages and γδT cells. While macrophages can phagocytose pathogens and produce cytokines and nitric oxide (NO) [38], γδT cells can rapidly produce effector cytokines to recruit other cells to the site of infection as well as exert cytotoxicity [39]. Measuring responses of these cell-types can aid in evaluating adjuvant-mediated effects in vaccinated chickens. The present study observed that CpG-ODN (10 μg) + rHVT-LT vaccination led to a significantly increased splenic populations of KUL-01+ macrophages and TCRγδ+ T cells, suggesting an adjuvant effect in enhancing innate immune response. Similar *in ovo* administered CpG DNA-mediated enhancement of macrophage responses in day-old chicks post-hatch has been previously reported [15,20,40,41]. These studies also suggested that CpG DNA-induced macrophage responses can contribute to an overall immune defense against ILT [20], IB [21], and avian influenza (AI) [42] viruses through production of pro-inflammatory cytokines and NO. To this end, the present study observation of increased transcription of IL-1β, IFNγ, and iNOS (a critical enzyme for NO production) in the spleen and lung of CpG-ODN-10µg + rHVT-LT-vaccinated chickens, suggests that the adjuvant effects not only enhanced macrophage frequency and tissue recruitment, but also may have augmented their production of immune mediators [20,21,40,42]. Our immunophenotyping analysis also found two distinct populations of KUL-01+ cells, of which both KUL-01 hi and KUL-01 Lo populations were found to be significantly higher in the CpG-ODN (10 μg) adjuvant vaccinated chickens. A previous study also noted a similar distinction in the KUL-01+ cell population while investigating anti-AIV responses [43], and furthermore, another study indicated that KUL-01 hi cells possess enhanced phagocytic and tissue migratory activity in chickens [44]. However, it remains to be investigated if these two phenotypically distinct populations of macrophages induced in response to CpG-ODN stimulation have any biologically distinct functions in chickens. In the context of present study observation of enhanced splenic γδT cell frequencies in the adjuvanted vaccine group, previous reports have also highlighted an important role for this cell-type in defense against viruses in chickens [45,46]. For example, we have previously shown that chickens infected with MDV had significantly higher number of splenic γδT cells and that these cells expressed elevated levels of IFNγ, suggesting their important role in antiviral defense [46]. Collectively, our observations show that CpG-ODN can effectively boost innate cell responses in response to *in ovo* administered rHVT-LT vaccination in chickens.

An inflammatory response to the immunizing agent or a virus infection is critical in the initiation of antigen-specific adaptive response and is often attributed to the innate immune cells, specifically the macrophages. While cytokines such as IL-1β and antimicrobial molecules such as NO can mediate inflammatory response in tissues, the regulation of inflammation between the local and systemic tissues also perhaps plays a critical role in the disease pathogenesis and antiviral defense. One of the findings from the present study was that CpG-ODN-mediated induction of IL-1β and iNOS genes was spatially regulated with a splenic expression of higher IL-1β and lower iNOS, while the lung expression was of lower Il-1β and higher iNOS genes. Although the biological significance of this effect needs further investigation, it seems reasonable to suggest that such a response may be derived from functionally distinct subset of macrophages induced in response to vaccination that vary in their tissue-specific inflammatory/regulatory activities [44] or perhaps, induction of regulatory T cells is locally at play. Another intriguing finding from the present study was that rHVT-LT vaccination ± CpG-ODN induced transcriptional downregulation of TLR21 in both spleen and lung tissues, yet the adjuvant effects were clearly evident throughout the analyses of the study. One or both of the following possibilities can be suggested: (1) the HVT virus (vaccine vector) may have evolved an immunoevasive strategy of downregulating TLR21 and possibly other virus-sensing receptors as in the case of other viruses, including herpesviruses [47,48] and (2) CpG-ODN-binding of TLR21 endosomally for the downstream MyD88 signaling may have reduced the receptor transcriptional abundance. Similar downregulation of TLR4 following to LPS stimulation of human monocytes has been previously reported [49]. Further studies in our laboratory investigating the transcriptional changes in the TLR21 expression at later timepoints post-hatch in the tissues of chickens administered in ovo with rHVT-LT vaccine ± CpG-ODN (10 µg) are currently underway. 

In conclusion, the present study showed that CpG-ODN (TLR21 agonist) can be used as an adjuvant for *in ovo* administration to boost rHVT-LT vaccine-induced immune responses in chickens. The immune parameters indicating adjuvant effects included increased expression of IFNγ, IL-1β and iNOS genes in the lung and spleen tissues coupled with an enhanced splenic macrophage, γδT and activated CD4+ T-cell responses.

## Figures and Tables

**Table 1:** Primer sequences used in real-time PCR.

Gene	Primer Sequence (F-Forward; R-Reverse)	Annealing Temperature (°C)	GenBank Accession Number
IFNγ	F: 5′-ACACTGACAAGTCAAAGCCGCACA-3′ R: 5′-AGTCGTTCATCGGGAGCTTGGC-3′	60	X99774
IFNß	F: 5′-CGTGTGCGAGAACAGCATGGAGA-3′ R: 5′-TCAGGCATTTCTCCTCGTCGAAGC-3′	60	NM_204628.1
IL-1ß	F: 5′-AGCAGATCAAGGAGACGTTC-3′ R: 5′-ATCAGCAGGTACTCCTCGAT-3′	55	AJ621614
IL-12	F: 5′-CCAAGACCTGGAGCACACCGAAG-3′ R: 5′-CGATCCCTGGCCTGCACAGAGA-3′	64	AY262752.1
TLR21	F: 5′-CCTGCGCAAGTGTCCGCTCA-3′ R: 5′-GCCCCAGGTCCAGGAAGCAG-3′	64	NM_001030558.1
iNOS	F: 5′-CCTGGTGATGCTGTGAATTG-3′ R: 5′-CTTCTGTGTCGTTGCATTCAG-3′	58	NM_204665
β-actin	F: 5′-CAACACAGTGCTGTCTGGTGGTA-3′ R: 5′-ATCGTACTCCTGCTTGCTGATCC-3′	58	X00182

**Table 2:** Chick hatchability following *in ovo* administration of vaccine ± CpG-ODN.

Treatment Groups	Number of Embryonated Eggs Inoculated	% Hatch
Sham	15	86.7
rHVT	15	86.7
rHVT + CpG-ODN 5 μg	15	93.3
rHVT + CpG-ODN 10 μg	15	80.0

## Data Availability

Research data available upon request from R.R.K.
